# Supplementary material for: HIVprotI: an integrated web based platform for prediction and design of HIV proteins inhibitors
Source: J Cheminform. 2018 Mar 9;10:12. doi: 10.1186/s13321-018-0266-y (PMC5845081; doi:10.1186/s13321-018-0266-y)
Supplement: Supplementary file 2 — Additional file 2. Source code of HIVProtI web server. [file 13321_2018_266_MOESM2_ESM.zip › HIVprotI_Source-code/hivprotI/weka-3-6-6/doc/weka/filters/unsupervised/attribute/SwapValues.html]

SwapValues


---


|  |  |  |
| --- | --- | --- |
| **Overview** \| **Package** \| **Class** \| **Tree** \| **Deprecated** \| **Index** \| **Help** \| **Weka's home** | |  |
| **PREV CLASS**   **NEXT CLASS** | **FRAMES**    **NO FRAMES**     **All Classes** |  |
| SUMMARY: NESTED \| FIELD \| CONSTR \| METHOD | DETAIL: FIELD \| CONSTR \| METHOD |  |


---


## weka.filters.unsupervised.attribute Class SwapValues

```
java.lang.Object
  weka.filters.Filter
      weka.filters.unsupervised.attribute.SwapValues
```

**All Implemented Interfaces:**: java.io.Serializable, CapabilitiesHandler, OptionHandler, RevisionHandler, StreamableFilter, UnsupervisedFilter

---

``` public class SwapValues extends Filter implements UnsupervisedFilter, StreamableFilter, OptionHandler ```

Swaps two values of a nominal attribute.


Valid options are:

```
 -C <col>
  Sets the attribute index (default last).
```

```
 -F <value index>
  Sets the first value's index (default first).
```

```
 -S <value index>
  Sets the second value's index (default last).
```

**Version:**
:   $Revision: 5547 $

**Author:**
:   Eibe Frank (eibe@cs.waikato.ac.nz)

**See Also:**: Serialized Form

---

**Constructor Summary**

`SwapValues()`


| **Method Summary** | |
| --- | --- |
| `java.lang.String` | `attributeIndexTipText()` |
| `java.lang.String` | `firstValueIndexTipText()` |
| `java.lang.String` | `getAttributeIndex()`             Get the index of the attribute used. |
| `Capabilities` | `getCapabilities()`             Returns the Capabilities of this filter. |
| `java.lang.String` | `getFirstValueIndex()`             Get the index of the first value used. |
| `java.lang.String[]` | `getOptions()`             Gets the current settings of the filter. |
| `java.lang.String` | `getRevision()`             Returns the revision string. |
| `java.lang.String` | `getSecondValueIndex()`             Get the index of the second value used. |
| `java.lang.String` | `globalInfo()`             Returns a string describing this filter |
| `boolean` | `input(Instance instance)`             Input an instance for filtering. |
| `java.util.Enumeration` | `listOptions()`             Returns an enumeration describing the available options. |
| `static void` | `main(java.lang.String[] argv)`             Main method for testing this class. |
| `java.lang.String` | `secondValueIndexTipText()` |
| `void` | `setAttributeIndex(java.lang.String attIndex)`             Sets index of the attribute used. |
| `void` | `setFirstValueIndex(java.lang.String firstIndex)`             Sets index of the first value used. |
| `boolean` | `setInputFormat(Instances instanceInfo)`             Sets the format of the input instances. |
| `void` | `setOptions(java.lang.String[] options)`             Parses a given list of options. |
| `void` | `setSecondValueIndex(java.lang.String secondIndex)`             Sets index of the second value used. |

**Methods inherited from class weka.filters.Filter**

`batchFilterFile, batchFinished, filterFile, getCapabilities, getOutputFormat, isFirstBatchDone, isNewBatch, isOutputFormatDefined, makeCopies, makeCopy, numPendingOutput, output, outputPeek, toString, useFilter, wekaStaticWrapper`

**Methods inherited from class java.lang.Object**

`equals, getClass, hashCode, notify, notifyAll, wait, wait, wait`

**Constructor Detail**

### SwapValues

```
public SwapValues()
```


**Method Detail**

### globalInfo

```
public java.lang.String globalInfo()
```

:   Returns a string describing this filter

    :   **Returns:**: a description of the filter suitable for displaying in the explorer/experimenter gui

---


### getCapabilities

```
public Capabilities getCapabilities()
```

:   Returns the Capabilities of this filter.

    :   **Specified by:**: `getCapabilities` in interface `CapabilitiesHandler` **Overrides:**: `getCapabilities` in class `Filter`
    :   **Returns:**: the capabilities of this object **See Also:**: `Capabilities`

---


### setInputFormat

```
public boolean setInputFormat(Instances instanceInfo)
                       throws java.lang.Exception
```

:   Sets the format of the input instances.

    :   **Overrides:**: `setInputFormat` in class `Filter`
    :   **Parameters:**: `instanceInfo` - an Instances object containing the input instance structure (any instances contained in the object are ignored - only the structure is required). **Returns:**: true if the outputFormat may be collected immediately **Throws:**: `UnsupportedAttributeTypeException` - if the selected attribute is not nominal or if it only has one value.: `java.lang.Exception` - if the input format can't be set successfully

---


### input

```
public boolean input(Instance instance)
```

:   Input an instance for filtering. The instance is processed
    and made available for output immediately.

    :   **Overrides:**: `input` in class `Filter`
    :   **Parameters:**: `instance` - the input instance **Returns:**: true if the filtered instance may now be collected with output(). **Throws:**: `java.lang.IllegalStateException` - if no input structure has been defined.

---


### listOptions

```
public java.util.Enumeration listOptions()
```

:   Returns an enumeration describing the available options.

    :   **Specified by:**: `listOptions` in interface `OptionHandler`
    :   **Returns:**: an enumeration of all the available options.

---


### setOptions

```
public void setOptions(java.lang.String[] options)
                throws java.lang.Exception
```

:   Parses a given list of options.


    Valid options are:

    ```
     -C <col>
      Sets the attribute index (default last).
    ```

    ```
     -F <value index>
      Sets the first value's index (default first).
    ```

    ```
     -S <value index>
      Sets the second value's index (default last).
    ```

    :   **Specified by:**: `setOptions` in interface `OptionHandler`
    :   **Parameters:**: `options` - the list of options as an array of strings **Throws:**: `java.lang.Exception` - if an option is not supported

---


### getOptions

```
public java.lang.String[] getOptions()
```

:   Gets the current settings of the filter.

    :   **Specified by:**: `getOptions` in interface `OptionHandler`
    :   **Returns:**: an array of strings suitable for passing to setOptions

---


### attributeIndexTipText

```
public java.lang.String attributeIndexTipText()
```

:   **Returns:**: tip text for this property suitable for displaying in the explorer/experimenter gui

---


### getAttributeIndex

```
public java.lang.String getAttributeIndex()
```

:   Get the index of the attribute used.

    :   **Returns:**: the index of the attribute

---


### setAttributeIndex

```
public void setAttributeIndex(java.lang.String attIndex)
```

:   Sets index of the attribute used.

    :   **Parameters:**: `attIndex` - the index of the attribute

---


### firstValueIndexTipText

```
public java.lang.String firstValueIndexTipText()
```

:   **Returns:**: tip text for this property suitable for displaying in the explorer/experimenter gui

---


### getFirstValueIndex

```
public java.lang.String getFirstValueIndex()
```

:   Get the index of the first value used.

    :   **Returns:**: the index of the first value

---


### setFirstValueIndex

```
public void setFirstValueIndex(java.lang.String firstIndex)
```

:   Sets index of the first value used.

    :   **Parameters:**: `firstIndex` - the index of the first value

---


### secondValueIndexTipText

```
public java.lang.String secondValueIndexTipText()
```

:   **Returns:**: tip text for this property suitable for displaying in the explorer/experimenter gui

---


### getSecondValueIndex

```
public java.lang.String getSecondValueIndex()
```

:   Get the index of the second value used.

    :   **Returns:**: the index of the second value

---


### setSecondValueIndex

```
public void setSecondValueIndex(java.lang.String secondIndex)
```

:   Sets index of the second value used.

    :   **Parameters:**: `secondIndex` - the index of the second value

---


### getRevision

```
public java.lang.String getRevision()
```

:   Returns the revision string.

    :   **Specified by:**: `getRevision` in interface `RevisionHandler` **Overrides:**: `getRevision` in class `Filter`
    :   **Returns:**: the revision

---


### main

```
public static void main(java.lang.String[] argv)
```

:   Main method for testing this class.

    :   **Parameters:**: `argv` - should contain arguments to the filter: use -h for help


---


|  |  |  |
| --- | --- | --- |
| **Overview** \| **Package** \| **Class** \| **Tree** \| **Deprecated** \| **Index** \| **Help** \| **Weka's home** | |  |
| **PREV CLASS**   **NEXT CLASS** | **FRAMES**    **NO FRAMES**     **All Classes** |  |
| SUMMARY: NESTED \| FIELD \| CONSTR \| METHOD | DETAIL: FIELD \| CONSTR \| METHOD |  |


---
